# Evidence-Based Cutoff Threshold Values from Receiver Operating Characteristic Curve Analysis for Knee Osteoarthritis in the 50-Year-Old Korean Population: Analysis of Big Data from the National Health Insurance Sharing Service

**DOI:** 10.1155/2018/2013671

**Published:** 2018-01-08

**Authors:** Hyunseok Jee, Hae-Dong Lee, Sae Yong Lee

**Affiliations:** ^1^Frontier Research Institute of Convergence Sports Science (FRICSS), Yonsei University, 50 Yonsei-ro, Seodaemun-gu, Seoul 03722, Republic of Korea; ^2^Integrated Sports Science Research Laboratory (ISSRL), Yonsei University, Seoul, Republic of Korea; ^3^Department of Physical Education, College of Sciences in Education, Yonsei University, 50 Yonsei-ro, Seodaemun-gu, Seoul 03722, Republic of Korea; ^4^Yonsei Institute of Sports Science and Exercise Medicine (YISSEM), Yonsei University, 50 Yonsei-ro, Seodaemun-gu, Seoul 03722, Republic of Korea

## Abstract

We aimed to investigate the characteristics of patients with osteoarthritis (OA), using the data of all Koreans registered in the National Health Insurance Sharing Service Database (NHISS DB), and to provide ideal alternative cutoff thresholds for alleviating OA symptoms. Patients with OA (codes M17 and M17.1–M17.9 in the Korean Standard Classification of Disease and Causes of Death) were analyzed using SAS software. Optimal cutoff thresholds were determined using receiver operating characteristic curve analysis. The 50-year age group was the most OA pathogenic group (among 40~70 years, *n* = 2088). All exercise types affected the change of body mass index (*p* < 0.05) and the sex difference in blood pressure (BP) (*p* < 0.01). All types of exercise positively affected the loss of waist circumference and the balance test (standing time on one leg in seconds) (*p* < 0.01). The cutoff threshold for the time in seconds from standing up from a chair to walking 3 m and returning to the same chair was 8.25 (80% sensitivity and 100% specificity). By using the exercise modalities, categorized multiple variables, and the cutoff threshold, an optimal alternative exercise program can be designed for alleviating OA symptoms in the 50-year age group.

## 1. Introduction

According to the database (DB) of the Health Insurance Review and Assessment Service, the proportion of patients with diseases of the musculoskeletal system was 50% in 2003 and 71% in 2013. In particular, knee diseases were the 10th most common cause of hospitalization, involving costs of about 2500 USD per person and ranking second in terms of total medical expenses (http://www.hira.or.kr). In addition, medical treatments take an average of 8 days to complete. Furthermore, the number of patients with OA increases steadily and OA pathogenesis shows a pattern of occurring even in younger age groups, which necessitates the establishment of prognostic and treatment programs for knee OA.

Several trials to identify multiple causes of knee OA have been performed to establish individualized and specific treatments for curing OA symptoms [1, 2].

As many cases have an unspecified etiology (causality; i.e., OA has multiple causes such as knee anterior cruciate ligament, meniscus damage, and quadriceps muscle weakness), providing individualized solutions for OA is difficult [3–5]. Because the causality between knee damage and OA cannot be clearly identified and it is difficult to find solutions for OA (many factors lead to aggravation of knee OA), prevention of OA is considered the ideal option [6]. To reach a reasonable solution to this issue, understanding the various characteristics of OA from diverse aspects is important to determine related factors affecting the pathogenesis of the disease and to decide appropriate optimal cutoff threshold points for improving prognostic programs (e.g., exercise prescriptions).

Big data analysis of health information is possible, as health-care providers mandatorily register their patient's injury or disease information through the National Health Insurance Sharing Service (NHISS) for reimbursement of medical service costs in Korea. The NHISS DB provides standardized health and medical information from unilateral medical check records of the entire Korean population. This DB was electronically organized, digitalized, and formatted from 2002 to 2013.

To address the above-mentioned issues and provide beneficial information about patients with OA for ameliorating OA symptoms from various aspects by using NHISS big data, we first set the following research objectives.

In this study, we aimed to examine the most prevalent ages of patients with OA among the elderly Korean population and characterize various features of OA according to four different exercise types, in order to provide evidence-based proper cutoff points for ameliorating OA pathogenesis. We examined three hypotheses in this study, as follows:

1. There would be specific age ranges of pathogenic OA in the elderly Korean population.
2. Variables related to comprehensive and physical function will show the overall characteristics of OA in relation to different exercise modalities.
3. There are appropriate cutoff thresholds for variables of pathogenic OA, and these can provide beneficial information for designing a reasonable prognostic program for improving OA symptoms.

## 2. Materials and Methods

### 2.1. Study Design

**Figure 1:**
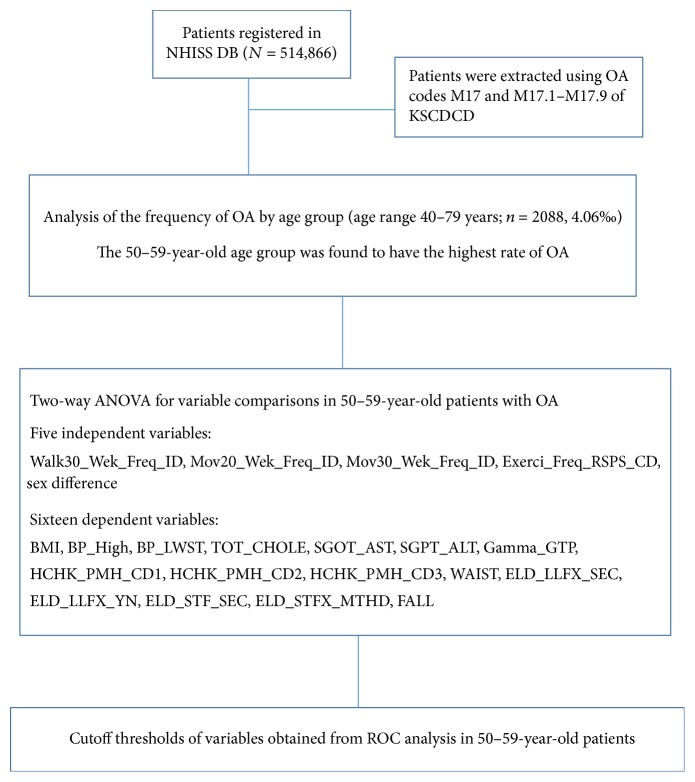
Diagram of the study design. A total of 514,866 patients enlisted in the NHISS were extracted. Patients with OA from the NHISS DB were subtracted by using KSCDCD-provided OA codes (M17, M17.1, M17.2, M17.3, M17.4, M17.5, M17.6, M17.7, M17.8, and M17.9), and patients with OA in this stage were those registered in the NHISS with a one-time diagnosis of OA. The age range of patients used to analyze the most OA pathogenic group was from 40 to 70 years. The effect of 4 independent variables (Walk30_Wek_Freq_ID, Mov20_Wek_Freq_ID, Mov30_Wek_ID, and Exerci_Freq_RSPS_ID) on 16 dependent variables (BMI, BP_High, BP_LWST, TOT_CHOLE, SGOT_AST, SGPT_ALT, Gamma_GTP, HCHK_PMH_CD2, WAIST, ELD_LLFX_SEC, ELD_LLFX_YN, STF_SEC, ELD_STFX_MTHD, and FALL) were verified by using univariate analysis of variance. Variables with statistical significance were selected and cutoff thresholds for optimal points were provided by using ROC curve analysis in 50-year-old patients with OA. OA, osteoarthritis; NHISS, National Health Insurance Sharing Service; KSCDCD, Korean Standard Classification of Disease and Causes of Death; Exerci_Freq_RSPS_CD, number of dates with exercise; Walk30_Wek_Freq_ID, number of dates with >30 min walking exercise in a week; Mov20_Wek_Freq_ID, number of dates with 20 min intensive exercise in a week; Mov30_Wek_Freq_ID, number of dates with 30 min moderate exercise in a week; BMI, body mass index; BP_High, maximal systolic blood pressure; BP_LWST, lowest diastolic blood pressure; ELD_LLFX_SEC, lower-extremity function assessing the time taken for walking 3 m away and back (only for 66 year olds); ELD_STFX_MTHD, balance test only for 66 year olds by taking the time of standing up with eyes closed or eyes opened; SGOT_AST, serum glutamic oxaloacetic transaminase and aspartate aminotransferase; SGPT_ALT, serum glutamate-pyruvate transaminase and alanine aminotransferase; TOT_CHOLE, total cholesterol.

This study is a retrospective cohort study of data from the NHISS DB. Korean Standard Classification of Disease and Causes of Death- (KSCDCD-) designated OA-related codes (M17 and M17.1–17.9) were used to identify patients with OA aged > 40 years from the NHISS DB. These patients were categorized into four age groups with increments of 10 years (40–49, 50–59, 60–69, and 70–79 years) to examine which age groups have a high risk of OA. The demographic, exercise, and functional test characteristics of each age group were subsequently investigated. The flow of the study is described in Figure 1.

### 2.2. Data Source and Subject Population

A total of 514,866 patients (approximately 10% of the whole population aged >40 years from the NIHSS DB pool) were randomly selected from patients registered in the NHISS. After the age of 40 years, Korean adults mandatorily need to undergo life cycle-based health checks, and the NHISS DB stores their data including exercise information and basic physiological health screening data (Table 1). An individualized design based on the NHISS data of patients aged > 40 years was used because OA-related symptoms with clinical evidence were observed at this age [7]. Patients with OA codes M17 and M17.1–M17.9 in the KSCDCD (http://kssc.kostat.go.kr/ksscNew_web/index.jsp) were extracted by using SAS software (Table 2). The KSCDCD defines M17 and M17.1 to M17.9 as indicating arthrosis of the knee. The study was approved by the institutional review board of Yonsei University (1040917-201603-HRBR-152-01E).

### 2.3. Categorization of Variables

Nineteen variables were divided into three categories. The first category comprised four exercise-related variables (Exerci_Freq_RSPS_CD: frequency of moderate-intensity exercise per week, Mov20_Wek_Freq_ID: frequency of 20 min intensive exercise per week, Mov30_Wek_Freq_ID: frequency of 30 min moderate-intensity exercise per week, and WLK30_Wek_Freq_ID: frequency of 30 min walking per week). The second category comprised 10 comprehensive variables including sex difference, body mass index (BMI, kg/m^2^), high blood pressure (BP_high, mm Hg), low blood pressure (BP_LWST, mm Hg), total cholesterol (TOT_CHOLE, mg/dL), serum glutamic oxaloacetic transaminase and aspartate aminotransferase (SGOT_AST, U/L), serum glutamic pyruvic transaminase and alanine aminotransaminase (SGPT_ALT, U/L), gamma glutamyl transpeptidase (Gamma_GTP, U/L), disease history (HCHK_PMH_CD2), and waist circumference (WAIST, cm). The third category comprised physical function-related variables such as lower-limb function test results for 66-year-old subjects (ELD_LLFX_SEC: time in seconds from standing up from a chair to walking 3 m and returning to the same chair and ELD_LLFX_YN: presence or absence of gait disability described as 1 or 2, respectively), balance test for 66-year-old subjects (ELD_STF_SEC: time of standing on one leg in seconds and ELD_STFX_MTHD: time in seconds of standing on one leg with eyes closed or open), and falls (FALL, fall injury within 6 months). The above-described variables were used to examine the second hypothesis and to provide ideal cutoff thresholds of statically examined variables of OA (hypothesis (3)) in the most OA pathogenic age group (hypothesis (1)).

### 2.4. Statistical Analysis

All data are presented as mean ± standard deviation. In the most OA pathogenic group, reciprocal effects and interactions were investigated by analyzing the established independent and dependent variables to understand the characteristics of OA. Statistically well-defined variables were then selected to provide the optimal cutoff thresholds in the most OA pathogenic patient group. As described in the* Categorization of Variables *section, the four exercise types were indicated as independent variables, and the 10 comprehensive and 5 physical function-related variables were considered dependent variables. Each of the four exercise types was tested for its effectiveness and interaction with the 15 dependent variables by using two-way univariate analysis of variance (ANOVA) in the 50-year OA group (to verify hypothesis (2)). By using logistic regression between patients with and without OA in the 50-year age group, risk factors according to the existence of OA were found and these variables were analyzed by using receiver operating characteristic (ROC) curve analysis to determine the cutoff points for the optimal threshold for avoiding OA pathogenesis (to examine hypothesis (3)). SAS version 9.4 (SAS Institute, Cary, NC, USA) and SPSS version 18.0 were used for all statistical analyses. A value of *p* < 0.05 was considered to indicate a statistically significant difference in all analyses.

## 3. Results

### 3.1. Subject Characteristics

All Koreans are mandatorily registered in the NHISS for their health check. A total of 514,886 randomly selected subjects from the NHISS DB pool were used to represent the entire population aged > 40 years, which was estimated to comprise about 5 million Koreans. By using the provided NHISS DB, sex-different demographic characteristics were identified and the values (men versus women) were as follows: total analyzed population, 279,125 versus 235,740; average age, 56.66 versus 58.36 years; BMI, 23.99 versus 23.99 kg/m^2^; TOT_CHOLE, 196.47 versus 203.08 mg/dL; SGOT_AST, 28.36 versus 24.66 U/L; SGPT_ALT, 29.11 versus 21.72 U/L; and Gamma_GTP, 52.04 versus 22.86 U/L (Table 1). The KSCDCD-derived codes indicating knee OA were M17 and M17.1–M17.9. The number of extracted patients with knee OA was 2088 (men versus women: 723 versus 1365), which means the number comprising single patients who had only one medical prescription for knee OA during 2002–2013. The values of average age, BMI, TOT_CHOLE, SGOT_AST, SGPT_ALT, and Gamma_GTP in patients with OA showed sex differences (Table 2).

### 3.2. Pathogenesis of OA in Each Age Group

**Figure 2:**
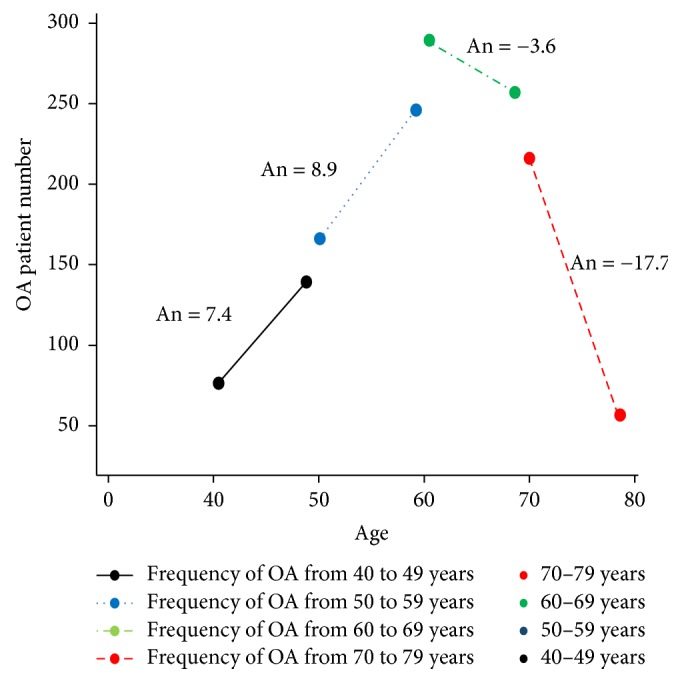
Pathogenesis of OA in each age group. Carefully extracted patients with OA who had only one hospital visit and OA prescription record in the NHISS from 2002 to 2013 were categorized into four age groups with increments of 10 years (40–49, 50–59, 60–69, and 70–79 years). Each angle from the linear functional graph in each age group was calculated to determine the increase or decrease of OA pathogenesis in patients. From this figure, the 50-year age group was selected and further investigated because the maximum OA occurrence was observed in this age group (An, 8.9). Compared with the 40- and 50-year age groups, the number of patients older than 60 years with OA pathogenesis unexpectedly decreased. OA, osteoarthritis; An, angle.

The subjects, aged from their 40s to 70s, were divided into four age groups with increments of 10 years (40–49, 50–59, 60–69, and 70–79 years). The linear function graph was verified to comparatively investigate the frequency of OA pathogenesis in each group (Figure 2). The angle of the graph for the 40–49- and 50–59-year age groups was 7.4° and 8.9°, respectively, and these groups showed an increasing OA pathogenesis pattern; however, the 60–69-year (angle, −3.6°) and 70–79-year (angle, −17.7°) age groups showed reversely decreasing values to patient numbers. Thus, we clarified our first hypothesis based on the observation that OA pathogenesis occurred most frequently in the 50-year age group.

### 3.3. Four Independent Variables (Exercise Modalities) on Comprehensive Variables

A sex difference was observed only in Walk30_Wek_Freq_ID according to age difference within 50~59-year age group (*p* < 0.01). All exercise types significantly affected the change of BMI; however, Walk30 showed a sex difference (*p* < 0.05). The interaction between sex difference and different exercise types was effective in changing BMI (*p* < 0.01). Sex difference affected BP in all exercise types (*p* < 0.01); however, exercise frequency did not seem to affect the change of BP in all exercise types. The four exercise types seemed more related to hypertension, cardiopathy, stroke, diabetes, cancer, and others than to tuberculosis, hepatitis, and hepatism in HCHK_PMH_CD2. Higher frequencies of exercise did not seem to affect the change of disease history; however, it depended more on the type of exercise (*p* < 0.01).

**Figure 3:**
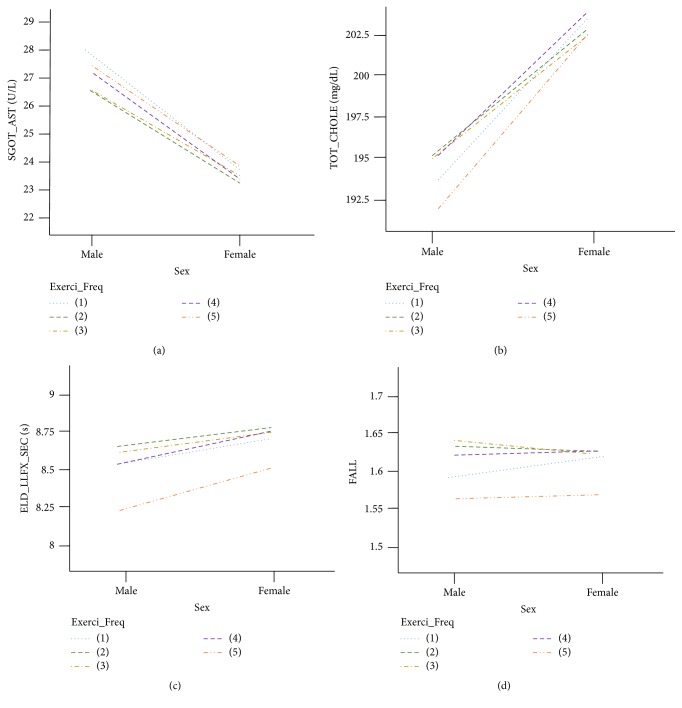
Comprehensive and physical function-related variables according to Exerci_Freq in 50-year-old patients with OA. Among 15 variables, (a) SGOT_AST (U/L), (b) TOT_CHOLE (mg/dL), (c) ELD_LLFX_SEC (s), and (d) FALL were representatively selected, as these variables had definite and similar patterns in Exerci_Freq among the four exercise types (Mov20_Wek, frequency of 20 min intensive exercise per week; Exerci_Freq, frequency of moderate-intensity exercise per week; Move30_Freq, frequency of 30 min moderate-intensity exercise per week; Walk30_Wek, frequency of 30 min walking per week). (1) do not exercise at all; (2) exercise 1-2 times per week; (3) exercise 3-4 times per week; (4) exercise 5-6 times per week; (5) almost daily exercise.

Male patients with OA aged 50~59 years had higher values of SGOT_AST and SGPT_ALT than female patients with OA. Higher frequency of exercise showed lower values of SGOT_AST and SGPT_ALT. Significant differences of sex and exercise type and interactions between sex difference and exercise type were found in SGOT_AST and SGPT_ALT (*p* < 0.01). The highest value of SGOT_AST was seen with the least frequency of male exercise. TOT_CHOLE in men was lower than that in women (*p* < 0.01). Only Exerci_Freq had no relation to TOT_CHOLE (*p* < 0.01). For improving TOT_CHOLE, Mov20 or Mov30, not Exerci_Freq, may be a better option.  Figure 3(b) shows that daily Exerci_Freq for improving TOT_CHOLE was more effective in men. All types of exercise significantly affected the changes of WAIST (*p* < 0.01).

Figures 3(a) and 3(b) show representative variables among 10 variables that had clearly common patterns according to the relationship to sex difference in Exerci_Freq.

### 3.4. Physical Function-Related Variables according to Independent Variables (Four Exercise Types)

All exercise types affected the changes in ELD_LLFX_SEC (*p* < 0.01), with patterns showing that daily exercise seemed to decrease the time in ELD_LLFX_SEC (Figure 3(c)). In ELD_LLFX_YN (presence or absence of gait disability), patients with OA with gait disability did not exercise at all, whereas all patients with OA without gait disability performed exercise. Sex difference was seen in all types of exercise (*p* < 0.01) except Mov20_Wek_freq. The balance test ELD_STF_SEC was affected by all exercise types (*p* < 0.01). Another balance test, ELD_STFX_MTHD, showed a significant difference in the effect of all exercise types (*p* < 0.05). Sex difference, exercise type, and the interaction between sex difference and exercise type had significant differences in FALL (*p* < 0.01).  Figure 3(d) shows that female patients with less exercise experienced falls more frequently, and this difference showed a decreasing tendency as the women exercised more (Figure 3(d)). A clear difference was observed between the daily exercise and less exercise groups (Figure 3(d)).

Panels (c) and (d) of Figure 3 represent similar patterns of each variable in the four exercise types.

We thus examined hypothesis (2) and concluded that there are specific high-risk dependent variables with the effect of exercise modalities in the incidence of OA.

### 3.5. Which Variables Affect OA Pathogenesis and What Are the Evidence-Based Optimal Cutoff Points of the Variables for Preventing and Alleviating OA Symptoms?

**Figure 4:**
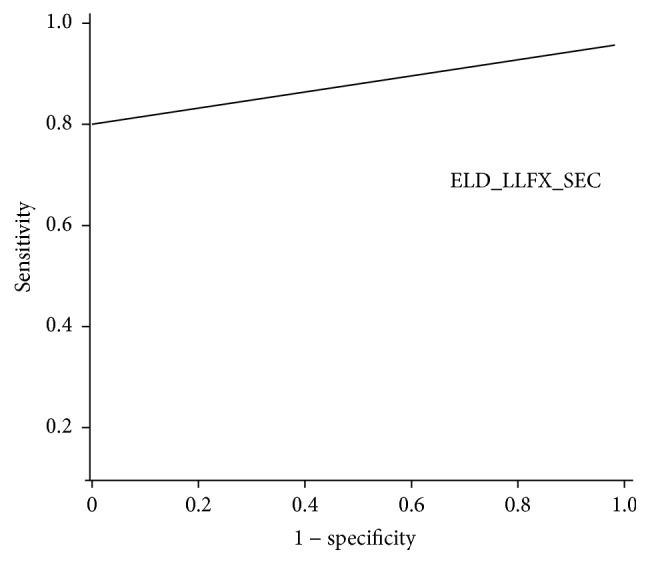
Graph of the ROC curve for the 50–59-year-old Korean population with knee OA. ROC curve analysis of ELD_LLFX_SEC (s) provided the optimal threshold value of 8.25 s, with area under the curve value of 0.861, sensitivity of 80%, and specificity of 100% (*p* < 0.01). ROC, receiver operating characteristic; OA, osteoarthritis; ELD_LLFX_SEC, time in seconds for standing up from a chair, walking 3 m, and returning to the same chair.

Randomly selected patients without OA registered in the NHISS were compared with patients with OA for logistic regression analysis. Among the variables, we found that FALL (*p* < 0.05) and ELD-LLFX-SEC (*p* < 0.01) significantly affected the pathogenesis of OA (Table 3). For 50-year-old patients with OA, ROC curve analysis provided ideal cutoff values for ELD_LLFX_SEC as 8.25 s (80% sensitivity and 100% specificity, area under the curve 0.861) (Figure 4). FALL did not have a meaning for the cutoff value because the responses for FALL were 1 (no experience of falls) or 2 (with experience of falls).

Hypothesis (3) was also clearly examined by using these results.

## 4. Discussion

We obtained the following OA-related results from patients registered in the NHISS by clustering the retrospective cohort of Koreans aged > 40 years.

1. OA pathogenesis was found most frequently in the 50-year age group.
2. The four analyzed exercise types affected the change of BMI (*p* < 0.05) and affected BP with a sex difference (*p* < 0.01); however, exercise frequency did not affect the change of BP. Exercise type and exercise frequency were important for SGOT and SGPT. Only Exerci_Freq did not affect the change of cholesterol (*p* < 0.01), and all exercise types affected loss of WAIST (*p* < 0.01). All types of exercise positively affected the balance test result (*p* < 0.01).
3. The optimal cutoff threshold for ELD_LLFX_SEC was 8.25 s (80% sensitivity and 100% specificity).

### 4.1. Big Data Analysis as a Useful Tool

NHISS is a worldwide unprecedented DB because all Korean citizens are mandatorily registered in this DB. Therefore, the entire medical history of the whole Korean population can be traced at any time [8].

Use of the NHISS DB overcomes the flaws of previous data sources such as private information and the limited contents of large-scale DBs. This DB allows researchers to access cross-sectional, retrospective, and prospective studies of each individualized patient derived from the DB cohort.

By using the large-scale NHISS DB, we were able to extract patients with OA according to the study design, and we were able to compare different age groups in terms of the pathogenicity of OA. In this study, we found that OA pathogenesis occurred most frequently in the 50-year age group among the randomly selected Koreans with OA aged > 40 years (*n* = 2088; nonoverlapping patients with only one prescription for OA) from the total cohort of 514,866 patients in the NHISS DB. Interestingly, the 60- and 70-year age groups were oppositely different from the 40- and 50-year age groups. The number of patients with OA in the 60–70-year age groups unexpectedly declined, and we presumed that patients in these age groups easily neglect visiting the hospital or have a higher mortality rate than the younger age groups, which affected our results. We believe that this DB will be useful in promoting national health and welfare.

### 4.2. Individualized Exercise Prescription for Reducing the Incidence of OA

A specific exercise prescriptive program based on these data can be designed. BP, cholesterol, and HCHK_PMH showed a pattern that is more related to the specific intensity, time, and frequency of exercise. The results showed that tailor-made exercise prescriptions are possible, and thus this study evidences that the DB can be an indispensable tool for improving the symptoms of OA. Our results comprehensively include microlevel to macrolevel data, from blood tests to physical function tests. Most modernized countries have emerging issues about experiences of falls in the aging society [9]. In Korea, 13% of 828 urban and 32% of 2295 rural elderly dwellers experienced falls in 1 year [9, 10]. The socioeconomic cost from fall injuries was calculated to be 343,614,988,000 Korean won [11]. To relieve this socioeconomic and individual burden, a more specially individualized exercise prescription can be an ideal option. According to this study, falls more often occurred in 50-year-old women with OA than in 50-year-old men with OA; thus, performing any kind of exercise on a daily basis can be an ideal option for improving fall symptoms in patients with OA.

### 4.3. Optimal Cutoff Threshold for OA Pathogenesis

The finding that OA occurred most frequently in the 50-year age group was used in further analysis to identify factors influencing the pathogenesis of the disease. We found that FALL and ELD_LLFX_SEC (*p* < 0.01) were associated with the occurrence of OA in the 50-year age group, and we suggest that this is associated with the muscle strength of the lower limb [12] (Table 3).

The results of four static and dynamic physically functional tests were examined in this study. Interestingly, only ELD_LLFX_SEC was significantly associated with the 50-year age group of patients with OA but not the three other similar functional tests (*p* < 0.01). This suggests that physically dynamic weight bearing load (ELD_LLFX_SEC) rather than static weight bearing load (ELD_STF_SEC; ELD_STFX_MTHD) beneficially suppresses damage on the knee joints [13]. According to the provided cutoff value, an immediate lower-limb strengthening exercise program should be developed to improve OA symptoms [14].

In conclusion, we obtained the following multifaceted results from the large-scale analysis of the NHISS DB, which can help in developing evidence-based OA preventive methods such as individualized exercise prescription programs.

1. Pathogenic OA was found most frequently in the 50-year age group.
2. All four exercise types investigated affected the change of BMI (*p* < 0.05) and had a sex-different effect on BP (*p* < 0.01); however, exercise frequency did not affect the change of BP. Exercise type and exercise frequency were important for SGOT and SGPT. Exerci_Freq only did not affect the change of cholesterol (*p* < 0.01), and all exercise types affected loss of WAIST (*p* < 0.01). All types of exercise positively affected the balance test (*p* < 0.01).
3. The optimal cutoff threshold for SGOT_AST and ELD_STF_SEC was 18.75 (88% sensitivity and 85% specificity) and 14.75 (75% sensitivity and 100% specificity), respectively.

This large-scale DB study may be indispensable in describing the risk factors affecting the aggravation of knee damage, for example, from anterior cruciate ligament to OA, and may lead to the development of standard and individualized prognostic OA interventions in the most OA pathogenic period of life.

## Figures and Tables

**Table 1:** Characteristics of the NHISS DB-based population older than 40 years.

Parameters	Values	
	Men	Women
Total analyzed population	279,125	235,740
Average age (years)	56.66	58.36
Follow-up years (years)	2002–2013	
BMI (kg/m^2^)	23.99	23.99
Total cholesterol (mg/dL)	196.47	203.08
SGOT_AST (U/L)	28.36	24.66
SGPT_ALT (U/L)	29.11	21.72
Gamma_GTP (U/L)	52.04	22.86

The total analyzed population from the NHISS DB showed sex differences in demographic characteristics. NHISS DB, National Health Insurance Sharing Service database; BMI, body mass index; SGOT_AST, serum glutamic oxaloacetic transaminase and aspartate aminotransferase; SGPT_ALT, serum glutamic pyruvic transaminase and alanine aminotransaminase; Gamma_GTP, gamma glutamyl transpeptidase.

**Table 2:** Characteristics of the NHISS DB-derived population with OA.

Parameters	Values	
	Men	Women
Total patients with OA	723	1365
Average age (years)	54.51	54.73
BMI (kg/m^2^)	24.42	25.50
Total cholesterol (mg/dL)	199.84	211.37
SGOT_AST (U/L)	31.06	25.81
SGPT_ALT (U/L)	29.97	23.83
Gamma_GTP (U/L)	63.85	23.21

Patients with KSCDCD-derived codes M17 and M17.1–M17.9 were extracted from the NHISS DB. NHISS DB, National Health Insurance Sharing Service database; KSCDCD, Korean Standard Classification of Disease and Causes of Death; OA, osteoarthritis; BMI, body mass index; SGOT_AST, serum glutamic oxaloacetic transaminase and aspartate aminotransferase; SGPT_ALT, serum glutamic pyruvic transaminase and alanine aminotransaminase; Gamma_GTP, gamma glutamyl transpeptidase.

**Table 3:** Logistic regression and ROC curve analyses for patients with OA.

Parameter	OR	AUC	*p* value	Cutoff values	Sensitivity (%)	Specificity (%)
Sex	1.973	0.559	<0.01	N/A	N/A	N/A
Age (years)	0.895	0.265	<0.01	N/A	N/A	N/A
SGOT_AST (U/L)	1.014	0.463	<0.01	18.75	88%	85%
ELD_STF_SEC (s)	0.189	0.022	<0.01	14.75	75%	100%
Fall	0.963	0.895	<0.01	N/A	N/A	N/A
ELD_LLFX_SEC (s)	0.478	0.861	<0.01	8.25	80%	100%

Variables with significant *p* values were selected via logistic regression analysis, and then other factors such as AUC, *p* value, and sensitivity and specificity of the cutoff points were considered to finally select the cutoff threshold values of the significant variables. ROC, receiver operating characteristic; OA, osteoarthritis; OR, odds ratio; AUC, area under the curve; N/A, not applicable; SGOT_AST, serum glutamic oxaloacetic transaminase and aspartate aminotransferase; ELD_STF_SEC, standing time on one leg in seconds; ELD_LLFX_SEC, time in seconds from standing up from a chair to walking 3 m and returning to the same chair.
